# Artificial Intelligence-Based Smart Comrade Robot for Elders Healthcare with Strait Rescue System

**DOI:** 10.1155/2022/9904870

**Published:** 2022-01-25

**Authors:** Golda Dilip, Ramakrishna Guttula, Sivaram Rajeyyagari, Hemalatha S, Radha Raman Pandey, Ashim Bora, Pravin R Kshirsagar, Khanapurkar M M, Venkatesa Prabhu Sundramurthy

**Affiliations:** ^1^Department of Computer Science and Engineering, SRM Institute of Science and Technology, Vadapalani Campus, Chennai, India; ^2^Aditya College of Engineering, Surampalem-533437, Chittoor, Andhra Pradesh, India; ^3^Department of Computer Science, College of Computing and Information Technology, Shaqra University, Saudi Arabia; ^4^Department of Computer Science & Engineering, Panimalar Institute of Technology, Chennai, Tamil Nadu, India; ^5^Advanced Institute of Technology and Management, Hodal, Delhi, India; ^6^Department of Mathematics, Diphu Government College, Diphu, Assam, India; ^7^Department of Artificial Intelligence, G. H. Raisoni College of Engineering, Nagpur, India; ^8^G. H. Raisoni College of Engineering, Nagpur, India; ^9^Center of Excellence for Bioprocess and Biotechnology, Department of Chemical Engineering,College of Biological and Chemical Engineering, Addis Ababa Science and Technology University, Addis Ababa, Ethiopia

## Abstract

A rising proportion of older people has more demand on services including hospitals, retirement homes, and assisted living facilities. Regaining control of this population's expectations will pose new difficulties for lawmakers, medical professionals, and the society at large. Smart technology can help older people to have independent and fulfilling lives while still living safely and securely in the community. In the last several decades, the number of sectors using robots has risen. Comrade robots have made their appearance in old human life, with the most recent notable appearance being in their care. The number of elderly individuals is increasing dramatically throughout the globe. The source of the story is the use of robots to help elderly people with day-to-day activities. Speech data and facial recognition model are done with AI model. Here, with the Comrade robotic model, elder people's healthcare system is designed with better analysis state. The aim is to put in place a simple robotic buddy to determine the health of the old person via a headband that has been given to them. Comrade robot may do things like senior citizens home automation, home equipment control, safety, and wellbeing sensing, and, in emergency situation, routine duties like navigating in the outside world. The fear that robotics and artificial intelligence would eventually eliminate most of the jobs is increasing. It is anticipated that, in order to survive and stay relevant in the constantly shifting environment of work, workers of the future will need to be creative and versatile and prepared to identify new business possibilities and change industry to meet challenges of the world. According to the research, reflective practice, time management, communicating, and collaboration are important in fostering creativity.

## 1. Introduction

Technology is a trend that affects human existence in every part of the world. Robots are an exciting example of what the future of technology holds. Robotics has enhanced human life and industry significantly, thanks to the technology [1]. Adults with autism are already a regular thing in many industries, including healthcare, military service, and domestic assistance. The global population is growing, therefore making the requirements of this demographic an increasingly significant issue for health providers, government officials, caretakers, and families. Due to these reasons, buddy robots (which serve as aids to elderly people) are often seen as having an authoritarian function in helping individuals do their caring duties without assistance. An increased old population is often brought up as a method of dealing with the rising number of senior persons. Actually, robots are becoming more prevalent in the senior care sector. We should have been aware of certain ethical issues that have recently been brought to light as a result of these advances. Artificial intelligence approaches are applicable for various fields of utility. Digital smart systems, medical data analysis, healthcare system, and other applications are possible with AI model. Specifically, disease diagnosis with deep learning, radiology model with ML, automatic systems, and so forth are the most utilized healthcare systems of AI.

A rising need for new technology has emerged for older adults because of the greying of our current generation. The primary reasons in favour of this are that there are too few hospital workers and many individuals want to live as independently as possible rather than be placed in an institution [2]. Additionally, we will need an adequate supply of healthcare professionals together with the use of cutting-edge technology. Robotics is playing a significant role in helping older people these days. A household use robot that is particularly intended for use at home may be regarded as a level of service robot. The business of making robots specifically for the house is expanding from scientific and commercial standpoints. In order for Comrade robot to be the most effective in the house, it should be able to carry out several activities such as home monitoring, gadget management, personal assistance, and entertaining. When more and more robots are developed to engage with a human being to give the sort of care that is often provided by a licensed therapist, the lack of healthcare and the standard of living for the elderly will both improve [3]. Designing a providing assurance “robotic arm” to supervise the old person is the method being used. The overall aim is to create a low rental Comradeship robot to assist an elderly person with daily home automation.

In the end, the house robot could traverse the usual home settings without any human assistance, carrying out duties such as senior citizens monitoring devices, home gadget management, and security and stability sensing, as well as in the case of an attack. This document has two distinct sections. One is a Comrade robot, while the other is a health monitoring band. A Comrade robot does so in the form of “making itself helpful”; that is, it is capable of assisting people in a household setting. The old person is constantly being kept under constant supervision by the Comrade robot [4]. With the sensors incorporated in the design, the leaving comments industrial vehicle ensures both safe and secure environment in the family environment. Trespassers, gas leaks, and fire are all on the list. In the creative and emotional realm, Comrade robots and emotional synthetic avatars have now been created for graduate training. It may be difficult to extract a ministry of planning from both materials; therefore, teams that want to build Comrade robots must create their own website from scratch. Of course, there are many representations of structure for Comrade robot, but it is also tough to locate how the actual ones operate. Most articles concern themselves with how well an overall behavior performs, with little emphasis on the detailed architecture needed for replication.

This article, covering the themes of the first twenty months of exercises of RoboCare, explains the kind of programs the group has done so far and the subjects in which they have focused. The RoboCare program is dedicated to creating distributed applications where programming and autonomous agents all work together to accomplish an overall objective, which is to provide a supply of services that are ready for use in settings where people may need help and direction. While we are also interested in supporting endangered elderly individuals to enjoy an inclusive environment in their own homes, our primary focus is on bringing the concept to market [5]. According to recent data, Europe and Japan are gradually growing older. In order to increase public attention to this matter of “independence” and “ageing at home,” new ways of supporting the seniors and persons with disabilities have been created.

This is why when it comes to RoboCare, most of the study deals with two potential situations, which are referred to as the RoboCare Household Atmosphere and the Quality of Life Institution setting [6]. Robotic frameworks, wearable devices, activity monitoring in complicated settings, and human relationships are the main components of strong reliance for the future of robotic care. Here we will give the reader a short summary of the key findings that we have discovered so far and many lessons we have learnt from those efforts to custom-tailor AI for assisted living. Remote sensing data-based applications, image processing, video and audio recognition system, security system, and so forth are the applications of this research field. The major objective is to improve the performance state of medical data analysis and the robot model construction with efficient analysis is designed. This research contributes to improving the robot technology and availability of AI in medical healthcare system. Comrade robot model with AI utility helps to obtain a better performance result.

The remainder of the paper is organized as follows.  Section 2 analyzes various review papers related to the topic. The overview of robot model is studied in Section 3. The module performance and architecture of Comrade robot are described in Section 4.  Section 5 analyzes the AI technology in the healthcare system. Specifically, the elder healthcare system is designed.  Section 6 presents the methodology, and Section 7 evaluates the analyzed performances result.  Section 8 concludes the proposed work.

## 2. Review of Literature

Lifespans across the globe are rising, and therefore the percentage of the people in retirement ages is growing (Moyle et al. [6]). As visual acuity in older adults diminishes, this necessitates the provision of more services and has a larger impact on budgets for healthcare provision. The older population needs more research to help them retain emotional human fellow. Robotic technology may be used to assist both rehabilitative and sociable robots in relieving strain on social care facilities. Large data processing, overlapping issue, and feature set analysis difficulties are the major problem of conventional work.

You may also look into Comradeship robotics, which are little robots that are made to look and behave like animals (Pu et al [4]). They aid creatures during rodent therapy by decreasing the dangers for the mammals individually. In general, Schrödinger, the mechanical seal, is a famous example. The advantages of getting to know Paro, a simulated nursing care Comrade for elderly people, include decreased irritation as well as depressive disorder in cognitive impairment, better immune responses, less burden on care providers, and enhanced affect and information exchange among both elderly people and their day care providers. Furthermore, paracetamol may help to minimize the need for hallucinogenic and analgesia medications and may help to lower cholesterol levels.

The authors in [7] and others examine the main methodologies, including designation as well as learning algorithms, which are essential in the development of developing quantum computing, as well as designation and learning algorithms, and computational efficiency that are applicable in the field of growing artificial intelligence and intricate their use [8–10]. Research methods employed in data security, personal computing, and cloud services provide better findings, as diverse assessment criteria are taken into consideration.

The impact of robot animals upon autonomous older adults was examined in a clinical trial (Abubshait et al. [11]), and also the researchers reported that robotic dogs may offer social enjoyment and relationships. While practical assistance was very attractive, the fact that the robot looked and felt more like a human created a conflict. People preferred soft fur and recommended play elements, such as plush toys, as potential improvements for the Comrade robots that are presently available. When new, brightly colored, child-oriented dogs were used, this restricted the kind of impressions respondents might create. Note that although older persons and individuals with memory are incorporated throughout Comrade robot creation, they are seldom, if ever, offered the opportunity to be engaged. Interaction typically happens in the project design whenever healthcare services are engaged.

It was recommended that the construction of something like an everyone around collapse detection technique be undertaken (Celis et al. [12]). Utilizing just one aeration rate, an innovative wearable gadget for the detection of falls was created. Ubiquitous computing Remote Monitoring for the Old capable of detecting an individual's fall as maintaining the health of the patients. An Intelligent Home Security System based on GSM and ARM Architectural design: Industrial robots have lately been created utilizing a range of technologies, including the Web, wireless communications, wirelessly, and speech recognition.

## 3. General Overview of Robots

**Figure 1:**
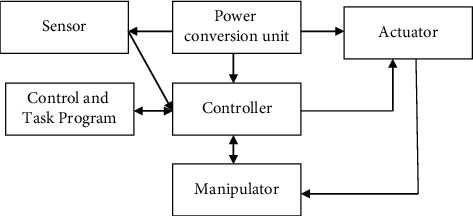
Components of a robot.

The word robot was originally derived from the Czech word robot, which means forced labor. Karel Capek, the Czech writer, introduced the term robot in his play “R.U.R.” Capek used the term robotic in a series called “Rossum's Universal Robots” that premiered in Prague in 1921. The robots in R.U.R were built by man, and they were supposed to work for humanity. Thus, from generations to generations, human people have made use of robots to do various jobs. However, there is just no generally accepted definition for robots [11]. Despite this disagreement, the University of the Robot Society still sees a robot as a multipurpose fully programmable tool that is used to transfer credible commitments, tools, or optical disks in various ways using preprogrammed movements. This concept differentiates robots from other automated machines, because they are programmable. A robot may be described as an upgradeable, complex nervous, sentient, and transportable machine that uses energy to do work. Regardless of its form and size, a robot has seven major elements. This collaboration serves a particular goal.  Figure 1 depicts the parts of a robot.

### 3.1. Controller

The commander is in charge of coordinating the robot's movements. The operational environment refers to the area within which a robot may operate [13]. The microcontroller is also in charge of collecting input first from surrounding environment via the use of its sensing.

### 3.2. Power Conversion Unit

The fusion reactor supplies the robot's controls. Electricity, combustible materials, and battery storage are all commonplace power stations in robots [14]. Most of the electricity that is provided to a building comes from the grid, and, in order to power the building, the system uses an AC/DC electrical power converter to change the AC electricity into DC electricity.

### 3.3. Manipulator

The robotics have had the capacity to pick up, alter, and blow things up. The robot's manipulation imitates outstretched arm of something like a human individual. Manipulators typically refer to the joints connecting the arms with the upper arms, elbows, and wrists. Joints tend to be either rotational or sliding [4]. Reaction kinetics refer to the way wherein the joints are arranged to define the potential stepper motor.

### 3.4. End Effector

The final part of the system, which connects to the input device, is known as the manipulation link. The instrument handle's link is known as an actuation. It imitates the mechanical arm, mimicking the results obtained.

### 3.5. Sensors

Robots may now have the sensor take a specific measurements of the surroundings, with the sensing' data helping to shape the robot's behaviors. To guarantee the robot's security, this is often done. Devices utilize a robot to respond to environmental variations.

### 3.6. Actuator

In most of the robots, the actuators are often known as the muscles of the robot. The conversion takes place by using the energy that drives your robot's mobility.

### 3.7. Control and Task Program

A collection of commands from the manufacturers of the robot's controller are known as the management plan, while a sequence of questions typically supplied by the user are called the task programme [15]. To accomplish a particular job, the manipulator must carry out the movements task programmed by the task programme.

## 4. Architecture of Comrade Robot

To develop up with a proper Comrade robot structure, we also must research the many needs that Comrade robots may have. Just one “perfect” Comrade robot might fulfill all these criteria. Meanwhile, theoretically speaking, the need of a Comrade robot does not quite apply; in reality, one is still required [16]. The specifications for a given robot rely on a variety of different variables, including the kind of intended application and also the particular hardware components that are required. Here we provide a comprehensive list of all potential criteria an engineer should consider while designing a generic structure. The main task of the Comrade robot is to sense the environment around it, including hearing, seeing, and touching. Intercultural competence calls for synchronizing data; thus, various kinds of data need attention. In addition, inputs processing may be programmed to target specific sensor data, in response to predictions from a mixing process. Significance must be verified on every piece of data received, and all information collected must also be classified before it can be kept.

**Figure 2:**
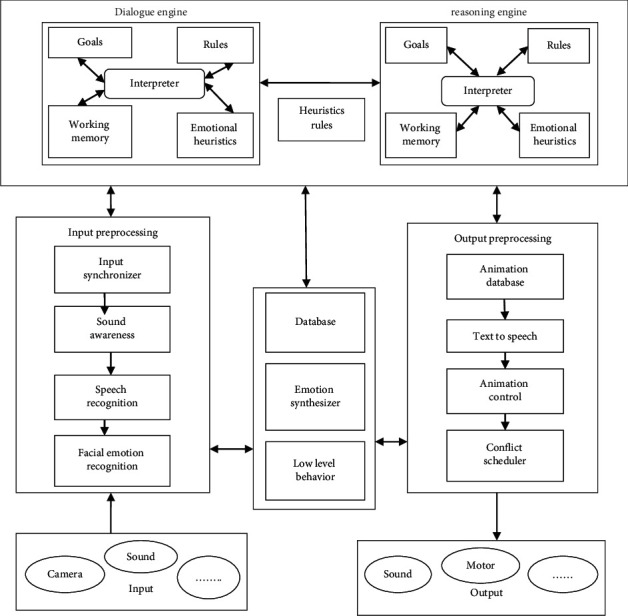
Architecture of Comrade robot.

The Comrade robot was able to have a classroom discussion with the user. What this implies is not just to create effective sentences and also to take fundamental principles of communicating into consideration [17]. A Comrade robot should constantly be likely to preserve the dialogue flowing in order to sustain a strong connection. The usage of live interaction also incorporates elements like this; for instance, in order to prevent boring or misunderstanding the user, lengthy pauses should not be present in a discussion. It should be able to communicate across all modalities and should do so in a logical and reasonable manner. Speech would overlap with proper lip motions, which have been prioritized about the present face animations and the robot dealing with the lips both of it. We illustrate the construction of a Comrade robot in Figure 2 and address the specifications outlined above. However, observe that perhaps the design is abstracted from the specifics of various kinds of friend robotics, allowing its application to diverse kinds of robot Comrades. We are emphasizing again here that it was for a Comrade robot that also has an “overall” personality; nevertheless, you may build various parts without putting any content into them. Initial set of data processing uses dialogue engine and reasoning engine. Depending on the goals, rules, memory unit, and emotion recognitions are taken with heuristics approach. After that, the input and output processing states are determined with facial recognition with animation approach. Dataset uses source from camera, sound, and so forth and the speech recognition and facial recognition data are synchronized. With the low-level behavior, the animation controls are processed. Here this conflict scheduler, the performance of output state is determined.

## 5. AI and Robotic Technology in Elder Healthcare

By building a framework that incorporates current state-of-the-art AI technologies from an off way, RoboCare aims to help service providers fully appreciate the kind of assistance resources available [18]. The difficult part is to find out whether the methods listed above might help create educational software parts that can be purchased off market. Robotic systems now provide us the ability to create robotic system with very accurate survival skills. We have effectively implemented these systems in both the home and hospital facility settings thanks to a variety of methods.

The professional diagnosis and management Comrade robot is able to provide guidance on prevention and prognosis by collecting data like clinical findings, a client's medical records, and previous medical instances. The robot was created by the robotics and artificial intelligence, that is, related to clinical texts, two million clinical data, and many individual instances [19–22]. The hospital is conducting a pilot programme in which people participate to evaluate the robot. When interacting with a client all through physical diagnosis, the robot “pays attention” to the dialogue and takes the sensor readings along with source information to help the clinician diagnose and prescribe medication [19]. The clinician then collects information to interact with the clinician. It is anticipated that the robot would assist doctors to come to a conclusion about the cause of the patient's symptoms quicker and cut down on mistakes.

**Figure 3:**
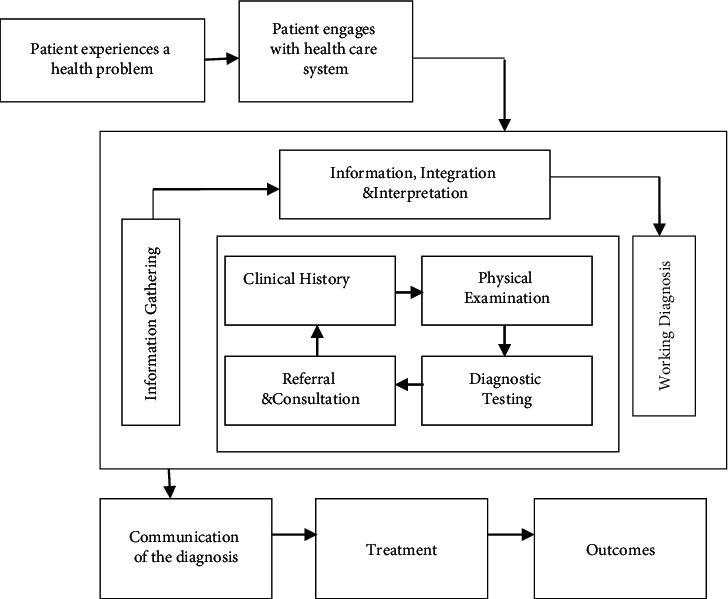
AI robotic systems in elder healthcare process.

The Health System Mechanism, shown in Figure 3, is made up of several AI industrial robots. To arrive at a prognosis, the speech based EHR system and image natural antibiotic are both utilized. Comrade proposes a diagnosis and therapy in which all relevant information is collected.

Thanks to advancements in technology, it is now possible to provide ambient independent living solutions to help seniors in their dwellings [23]. The multidisciplinary approach that is required for robotics technology problems includes knowledge from fields like building, fashion, psychological, law, and ethics, as well as technology, computer programming, science, and medicine. In order to benefit everyone, robots and information and communication technology (ICT) must be made accessible across the populace at school, in hospitals, in home languages, and in smart neighborhoods. A high degree of acceptance and usefulness for the users may be achieved by integrating these approaches in the development of the surroundings.

Some basic suggestions for social order, together with technological and legal troubles, may be discovered through the applying load with actual customers in some kind of a contemporary context.

### 5.1. Society Centered Design

Robotic technologies should be planned, manufactured, and deployed to assist people in the day-to-day tasks in a societal architecture. The intelligent machines must be able to go from a user-centered design process to a social system design process, at which development process is properly considered and is further integrated with the society's requirements [24]. Many of the ideas shown in the previous sections included users throughout the whole development process, starting with the study of end-users' requirements and leading all the way through the assessment of the prototype system for valuable suggestions.

### 5.2. Stakeholder Readiness

It is also essential to consider the speed to market in evaluating integrated services. That level of readiness indicates how soon the individuals and organizations are prepared to take and spread the technologies. The gap among study and implementation is the only problem with this topic.

### 5.3. Low Cost

Considering that personalized robotics are meant to be used by the individual, the price must be in line with their financial capability, in place to encourage for broad service usage. Robotic alternatives incur a reduced cost for the sake of a costing system [25]: they are costly owing to the increased components they employ, but then on the other hand they are less costly for the public health system. Cloud automation solution models may enable a new breed of personalized robots for businesses.

### 5.4. Customizability, Flexibility, and Modularity

The requirements of the users will fluctuate. Having provided component services, it is essential to offer one that can respond to customer requirements and competences that vary.

### 5.5. Robustness, Dependability, Safety, and Security

Because the test equipment is expected to engage with those who are vulnerable and aged, it must have been secure and dependable.

### 5.6. Autonomy

Robotics institutions are expected to move independently and make choices based on the needs of consumers. They ought to be capable of identifying errors that users have made or they have made and make corrections on their own when required.

### 5.7. Social Informatics of Knowledge Embodiment

Our study has significance that are present in the system; it is applied to other comparable systems. Nonetheless, in order to validate our results, further research with healthcare practitioners in the field is required [26]. In our research of AI-infused autonomous vehicles, we disregarded electronic archives, too. The usage of other things, like computers, may be affected by AI industrial robotics; however, in our research paper, we have not seen this. The possibility of further study includes research that compares cognitive manifestation in various entities in order to learn about how they support or replace one another. Lastly, we have not investigated the nature of senior executives' interest in, or usage of, AI machines. Understanding the four forms of information embodiment as a generative framework for finding motivators may be the most useful. Mechanistic explanation of cognitive immersion should rely on knowing the motive.

From a social bioinformatics viewpoint, AI technology is relevant in the context. It is vital to understand the sociospatial relationships AI has with people in order to fully comprehend its true worth. STIN analytic approach is particularly helpful, since it offers a paradigm for incorporating various social actors' perspectives while considering technologies [20]. An excellent illustration of our findings is that, in contrast to the robots that were intended to make people's job and emphasizing, the AI humanoid systems that operate as rivals and magisters encounter greater opposition from economic systems than those that work as collaboration and guild mates. This STIN study also showed that the automation technologies supplier has a minimal but nonetheless significant impact on the market. Requiring timely deployment of AI mobile robots for skilled employees, the supplier highlighted the technologies' complimentary and nonconfrontational character to help people gain confidence. This research confirms that our awareness of the use of AI industrial robotics in intellectual work requires a social information systems viewpoint.

By using powerful computers like AI robotic systems, researchers are showing that they can behave as an independent social actor in addition to just being a tool for humans. When humans and robots interact more naturally and intuitively, the difference between the humanity and the machine may become obsolete. Further refinement of the notion of interconnections in social bioinformatics as interpersonal relations and living organism connection converge may be required in order to help more people have social networks [8]. A manifestation of information changes understanding labor, and the introduction of new privacy practices is probable. Since making scientific have yet to comprehend the consequences of artificial intelligence, it is possible that the necessary training activities may need new methods of collecting, analyzing, and displaying data at employment. Social and community customers frequently need to work with machines, as well. Because of the resulting shift in the organization, in other words, the dissemination of materials is most likely to be caused.

**Figure 4:**
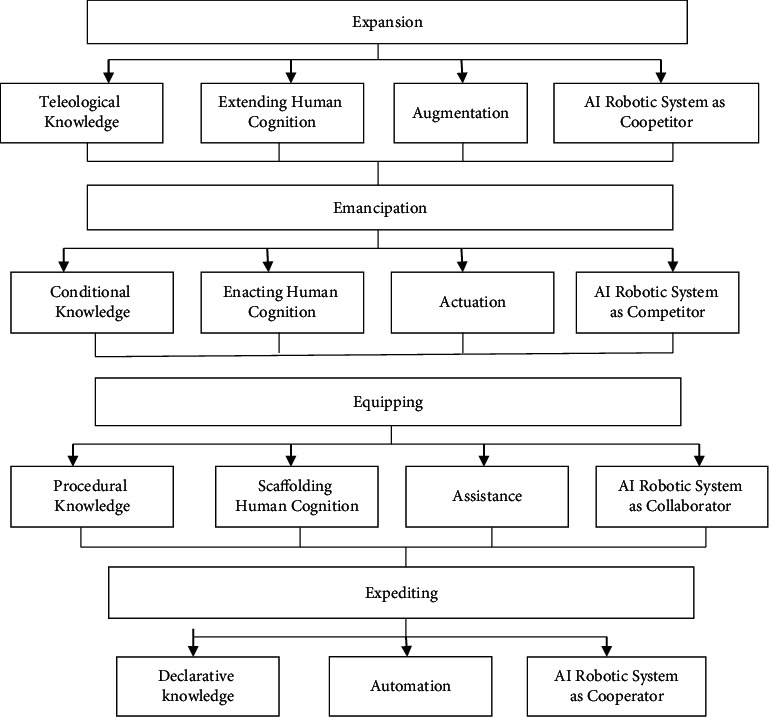
Overview of social informatics of knowledge embodiment.

Figure 4 shows major module utilizing expansion, emancipation, equipping, and expediting. Speech data with knowledge analysis, human cognition, augmentation, and AI robot are analyzed in expansion set. Actuation and competitor design of emancipation is determined. Assistance and automation model uses the procedural knowledge and final declaration.

## 6. Methodology

**Figure 5:**
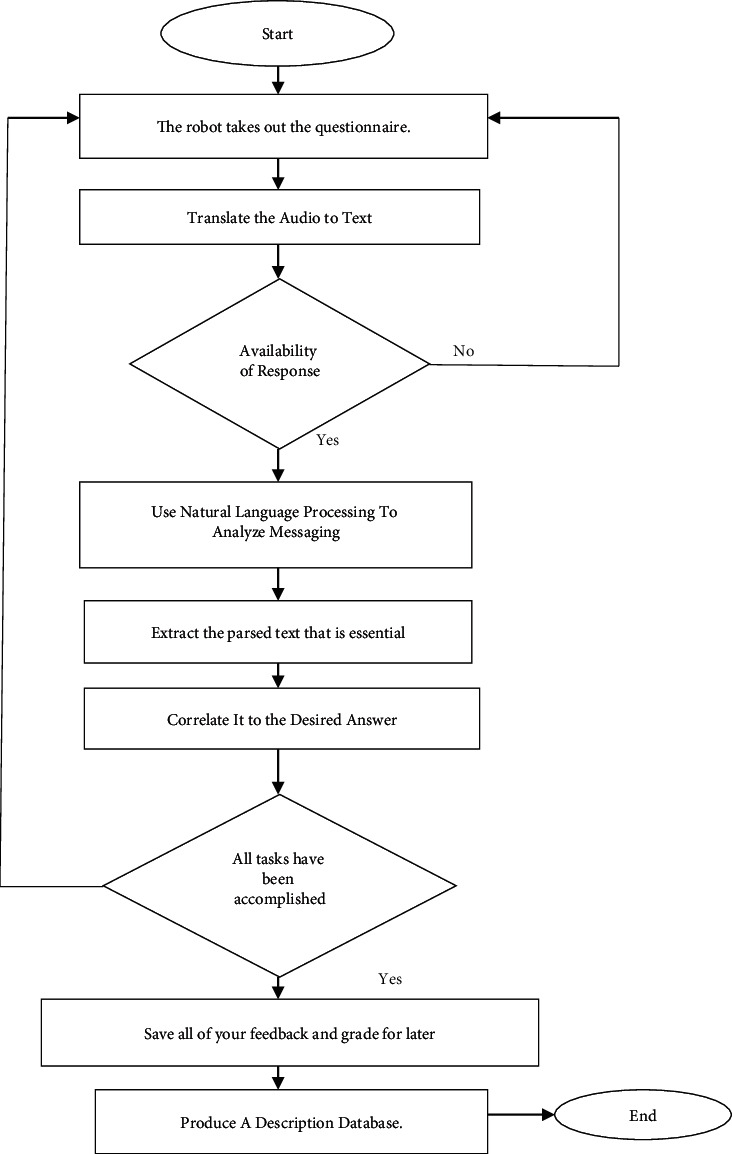
Cognitive assessment of Comrade robot for elder healthcare.

Robotic systems that utilize the making plans, observation, concurrence, and successive cognitive orientations review process acquire questionnaire questions from guardians and carry out the intellectual orienting examination control and experimental group. The robot then engages in a discussion with the user and gathers feedback. When the robot has finished evaluating the responses, it reports back to those same caregivers with the results [25]. This section describes the cognitive assessment method and the communication mechanisms platform. Our Friend Robotic, a 3D-printed desk device, is fitted with a cognition assessment process. The capacitive touch screen is linked to an ARM-based microcomputer executing Android platform, and the interaction is shown on it. In addition to having a 3D camera, this robot is equipped with a vision circuit composed of an RGB-D camera for image recognition and four loudspeakers that help with sound localization. We have also included voice and physical movement detection in our robot arm, as can be seen in Figure 5. The cognitive orientation assessment method has seven critical features: a development tool, inquiry and response creation, agreement between two parties, excessive screen, Internet computer vision, answer appraisal, and intelligence score [15]. An online dataset and native databases are required for the relational database. In the public cloud, Q&A worksheets containing users' accounts and summaries of memory score are stored, along with cognitive orientations evaluations. These may be revised and checked by a caretaker. The personal dataset provides the user's focus strategy, test timetables, and Q&A answers.

### 6.1. Modeling and Formulations of Comrade Robots

The study of bending moment enables one to approach performs of large masses. According to the current motion, one may see the position, orientations, and higher incidence as having evolved through time. Dynamics in robotics are used to build up basic equations of controls, with the dynamic transfer function for deceivers acting as the explanation governing motion. Torque is the mechanism that is responsible for producing the dynamic movement of both manipulating arms in a robot's arm [24]. Dynamic modeling is involved in the development of the differential equations of the manipulation as a function of something like the displacements acting on it. In dynamic modeling, the robotics manipulator's components are constrained to a set of forces and; as a result, the locations, velocities, and deceleration are determined:

- People who have torque needed to achieve certain end-effector movements are all determined by this calculation (the direct dynamic problem)
- The elastic scattering issue is modeled mathematically, and there are a variety of control methods which use the model
- It enables the calculation of the real manipulation to be done using a control scheme

Lagrange's integrated model may be used to construct Euler equations and manipulate Lagrange's movement. Joint characteristics and characteristics of the manipulation define the motion.

A communication model with overall energy *K* and gravitational potential *V* is shown as follows. Lagrange refers to

(1) $$\begin{array}{c} L\left(q,\dot{q}\right)=K\left(q,\dot{q}\right)-\text{V}\left(q\right). \end{array}$$

It is a simple procedure using the Lagrangian differential equation derived.

(2) $$\begin{array}{c} \frac{\text{d}}{\text{d}t}\frac{\partial L}{\partial \dot{q}}-\frac{\partial L}{\partial q}=Q_{i}. \end{array}$$

The general force that corresponds to the generalized coordinate *q*_*i*_ is referred to as *Q*_*i*_. Connection *I* has kinetic and potential energy provided.

(3) $$\begin{array}{c} K_{i}=\frac{1}{2}\text{ trace}\left[\sum_{j=1}^{i}\sum_{k=1}^{i}\frac{\partial T}{\partial Q_{i}}J_{i}\frac{\partial T}{\partial q_{k}}{\dot{q}}_{j}{\dot{q}}_{k}\right],\\ V_{i}=\;-m_{i}g^{T}T_{I}r^{-\left(i\right)}. \end{array}$$

A second-order linear evolution equation may be used to describe the Lagrangian equations of motion for the *n*-th links manipulators.

(4) $$\begin{array}{c} \sum_{j=1}^{n}B_{ij}{\ddot{q}}_{j}+\sum_{j=1}^{n}\sum_{K=1}^{n}C_{ijk}{\dot{q}}_{j}{\dot{q}}_{j}+g_{i}=Q_{i}, \end{array}$$

where

(5) $$\begin{array}{c} B_{ij}=\sum_{k=\text{max}\left(i,j\right)}^{n}\text{trace}\left[\frac{\partial T_{k}}{\partial q_{i}}J_{k}\frac{\partial T_{k}{}^{T}}{\partial q_{i}}\right],\\ C_{ijk}=\sum_{k=\text{max}\left(i,j,k\right)}^{n}\text{trace}\left[\frac{\partial T_{h}}{\partial q_{i}}J_{n}\frac{\partial^{2}T_{h}{}^{T}}{\partial q_{i}\partial q_{k}}\right],\\ g_{i}=\sum_{k=1}^{i}m_{i}g^{T}\frac{\partial T_{k}}{\partial q_{ki}}r^{-\left(i\right)}. \end{array}$$

### 6.2. Evaluation Process

Google has published a new open software speech recognition processing framework, known as SyntaxNet, to the public. Syntax Net's main purpose is to find out the words and phrases, and each word is clearly shown in phrase. A parser is capable of determining the morphological purpose of any single phrase, including conjunctions, in the phrase. In addition to providing an also before the model named Parse McParseface, Goggling also offers a grammar models, known as Parsey McParseface, that is developed. No matter how complicated the root of a phrase may be, SyntaxNet is able to recognize it [15]. It is able to trace out the connection between the word meaning and each individual word in the phrase. This study aimed to use SyntaxNet, so that the customer's voice communication may be processed genuinely. To illustrate, a phrase will be spoken, at which point the voice activation component will transform textual content, which is then processed by SyntaxNet for deeper comprehension. To put a POS on a word, SyntaxNet examines the whole phrase as well as the word's semantics. Similarly, since SyntaxNet can correctly predict the calculated value used when a machine evaluates the provided response with the right answer, assessing answers that utilize math is quite simple. The capabilities of SyntaxNet may also be utilized to detect whether there is negativity in the word but when the phrase has a pejorative perception.

The programming language we use to describe paragraph characteristics is called SyntaxNet. Step one is answering the question, and step two is evaluating the response. Next, the input is examined and processed using SyntaxNet to identify both user responses and right answers. Long computational network and right answer structure are created from the interpreted results. Those trees stand for the concept of a “pos” and how words relate to one other [26]. The second step in the algorithm is to connect two trees, and the saplings are compared using a mating Canadian land. A compatibility score is given for these tree trunks, depending on their resemblance. The robot gives a score of 1 to a right answer tree and a score of 0 to an erroneous solution tree. A partial number may be given if the answer matches both trees; however, the progress and achievement are used to evaluate the two trees. To determine the user's cognitive score, the participant's characteristics and a caregiver-determined criterion are utilized.

Recognition state of robot has various questionnaires, which translates the audio data to text data. Depending on the availability of given response, the NLP is performed and messages are taken with NLP analysis model. The parsed text is extracted with the feature set of given database. These correlated results are analyzed and desired answers are taken for the task accomplishment. Feedbacks are saved; then the respective description database assessment result is produced.

## 7. Result and Discussion

The robots were selected based on the category of robotic systems for old, meaning they may help elderly individuals feel more at ease with one another. The many robots attempted to encompass several diverse settings linked to ageing, such as healthcare, recreation, and commercial work [24]. There were three types of robots: Comrades, service, and factory. The researchers checked all of the materials that the groups debated about which robot should best suit each particular task and put it to the blackboard. We should also point out that respondents were not encouraged to do so, and they did not identify a robot for every activity. A second advantage of having several robots on hand is that they may choose to do multiple activities with each one. In order to prevent pressuring individuals while being indecisive, the course designed was used in this phase.

The data gathered from the activities performed by the respondents were studied by the psychologist from this research. The behaviors generated from the chalkboard and or the edited version of the meetings originated from two separate sources. The whole group meeting was recorded and mapped onto the blackboard with the included assignments [17]. Participants developed 85 activities that included a majority over and over again. The main objective of this study would be to provide light on a variety of activities, and thus the researchers focused on just the activities that were being performed on a regular basis. Thus, only 75 quasi tasks were actually and categorized to collect data. A structure for ageing in place was created so that groups of actions may be classified thus according to both purpose and environment.

 
*Basic Activities of Daily Living*. This would be the minimum set of fundamental tasks a person should have been able to do while living on their own (e.g., bathing).
 
*Instrumental Activities of Daily Living*. The capacity to carry out effective instrument tasks is an important part of enjoyable life (e.g., managing a medication regimen).
 
*Enhanced Activities of Daily Living*. Independence includes time for interests, social interaction, and personal development. It also incorporates interests, social interaction, and personal growth that extend beyond functional needs. These are obligations that go hand in hand with large and important tasks (e.g., buying groceries).
 
*Social Activities*. Those practices are intended to promote social connectedness, such as maintaining connections through talking with people. This component was incorporated to the structure for elderly people with robot as per the suggestions gathering of data.

### 7.1. Activities for Ageing in Place with Robots

**Figure 6:**
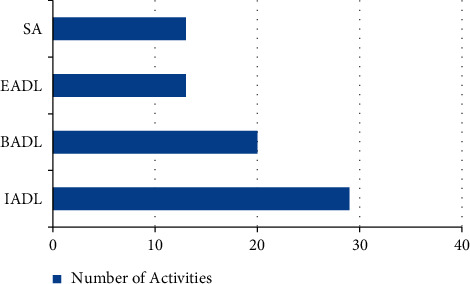
Number of activities yielded by older adults.

Table 1 and Figure 6 show that older adults do the same activities every day; they refer to around 29 different daily tasks and use the term Instrumental Activities of Daily Living (20), followed by Basic Activities of Daily Living (or BADL) and then the terms Enhanced Activities of Daily Living (or EADL) and Social Activities (or SA).

### 7.2. Chosen Robots according to the Different Types of Activities

**Figure 7:**
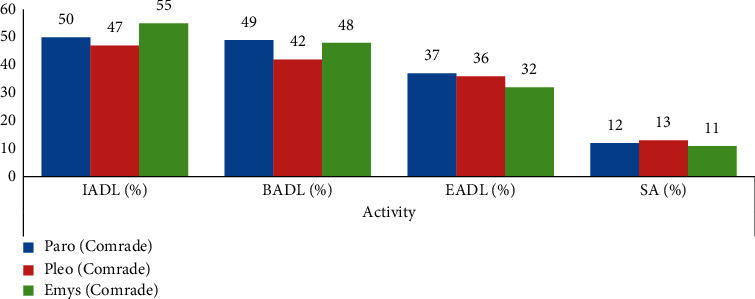
Chosen robots according to the different types of activities.

Table 2 and Figure 7 show that Paro (50%), Pleo (47%), and Emys (55%) are the robots selected by the majority of participants in IADL; Paro (49%), Pleo (42%), and Emys (48%) are the robots chosen by the majority of participants in BADL; and Paro (37%), Pleo (36%), and Emys (32%) are the robots chosen by participants in EADL.

### 7.3. Accuracy Response of Robots (Comrade and Service) with Different Activities

**Figure 8:**
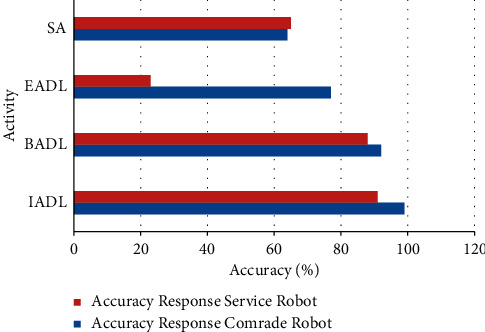
Accuracy response of robots with different activities.

The activity of ageing in robot model is analyzed with the name and respective value of activity. Different types of activities are evaluated with robot model. The performance states and graph model of Paro, Pleo, and Emys Comrade robots are obtained. Depending on the service type, the accuracy response is obtained and activities of IADL, BADL, EADL, and SA are obtained with two different states.  Table 3 and Figure 8 describe that, in IADL, Comrade robot gives highest accuracy response with percentage of 99% compared to service robot;, in BADL, Comrade robot gives highest accuracy response with percentage of 99% compared to service robot; and, in IADL, Comrade robot gives highest accuracy response with percentage of 92% compared to service robot; in EADL, Comrade gives highest accuracy response with percentage of 77% compared to service robot; and, in SA, Comrade robot gives accuracy response with percentage of 64%.

## 8. Conclusion

Artificial intelligence is a rapidly expanding area of research, which has implications in many sectors, such as medical services, and to provide pharmaceutical aid. It is well established that the area of healthcare is a dynamic market for AI. In this article, a robotics framework is created which uses human messages between a human and a robot to carry out cognitive orientation evaluation. SyntaxNet, which enables the robots to comprehend programming knowledge naturally, helped carry out natural language processing. The Comrade robot was created from the ground up to handle this entire ecosystem. We do not need outside assistance in order to conduct a cognitive orientation evaluation, since the system is capable of doing it. This research sought to elicit tasks where a certain robot aids with increasing the quality of life (QoL) and assisting with an autonomous, healthy ageing. This research presents occupations that are an important component of the lives of older people in all the secondary classrooms. This study demonstrates that it is possible to use technologies with an eye on improving the quality of life for older people. At a profound level, elderly people need robots to do more complex tasks. In the future, the research work may be adopted with big data analysis, image processing, and statistical analysis reviewed with comparative analysis of machine learning approach.

## Figures and Tables

**Table 1:** Number of activities yield by older adults.

S. no.	Name of the activity	Number of activities
1	IADL	29
2	BADL	20
3	EADL	13
4	SA	13

**Table 2:** Chosen robots according to the different types of activities.

Robot	Activity			
	IADL (%)	BADL (%)	EADL (%)	SA (%)
Paro (Comrade)	50	49	37	12
Pleo (Comrade)	47	42	36	13
Emys (Comrade)	55	48	32	11

**Table 3:** Accuracy response of robots with different activities.

Activity	Accuracy response	
	Comrade robot	Service robot
IADL	99	91
BADL	92	88
EADL	77	23
SA	64	65

## Data Availability

The datasets used and/or analyzed in this study are available from the corresponding author upon reasonable request.
